# Patients with enthesitis related arthritis show similar monocyte function pattern as seen in adult axial spondyloarthropathy

**DOI:** 10.1186/s12969-020-0403-9

**Published:** 2020-01-15

**Authors:** Shruti Bhattacharya, Ramnath Misra, Amita Aggarwal

**Affiliations:** Department of Clinical Immunology and Rheumatology, Sanjay Gandhi Postgraduate Institute of Medical Sciences, Lucknow, India

**Keywords:** Toll like receptors, Lipopolysaccharide, Monocytes, Inflammation, Juvenile idiopathic arthritis, Ankylosing spondylitis

## Abstract

**Background:**

Axial SpA and Enthesitis related arthritis (ERA) patients show strong HLA-B27 association, gut dysbiosis, high toll like receptor (TLR)2 and 4 expression on monocytes, pro-inflammatory cytokine production and elevated levels of TLR4 endogenous ligands [tenascin-c (TNC) and myeloid related protein (MRP)8/14] in serum. Hence, we aimed to understand if these diseases have similar or different monocyte response.

**Methods:**

Fifty adult axial SpA, 52 ERA patients and 25 healthy controls (HC) were enrolled. Cytokine-producing monocyte frequency before and after stimulation with lipopolysaccharide (LPS), peptidoglycan (PG), TNC or MRP8 were measured in whole blood (WB) and synovial fluid mononuclear cells (SFMC) by flow cytometry. Also, IL-6, TNF, MMP3, TNC and MRP8/14 levels were measured in unstimulated and TLR ligand stimulated WB cultures supernatant by ELISA. Finally, the mRNA expression levels of TNF and IL-6 were measured post stimulation with LPS, TNC and MRP8.

**Results:**

At baseline, ERA and axial SpA patients showed similar TNF-α producing monocyte frequency which was higher than HC. MRP8 simulation led to increased TNF-α producing monocyte frequency in ERA than axial SpA. TNC and MRP8 stimulation led to similar IL-6 producing monocyte frequency in axial SpA and ERA patients. Baseline TNF and IL-6 producing monocyte frequency also modestly correlated with disease activity scores. TNF and IL-6 producing monocyte frequency increased in response to TLR stimulation in SFMC from both patients.

In culture supernatants, axial SpA and ERA patients showed similar TNF production at baseline. MRP8 and TNC stimulation led to higher TNF production from ERA. Baseline IL-6 and MMP3 production was higher in ERA while TLR stimulation led to similar IL-6 and MMP3 production from axial SpA and ERA. TNC stimulation led to higher MMP3 production in ERA. mRNA expression in response to TLR stimulation was observed to be similar in axial SpA and ERA.

TNC production was higher in ERA at baseline, while MRP8/14 production was higher in axial SpA than ERA post stimulation.

**Conclusion:**

ERA patients have similar monocyte response to exogenous and endogenous TLR ligands as patients with axial SpA. This suggests that differences between pediatric and adult-onset SpA are minimal and they may have a common pathogenesis.

## Background

Spondyloarthropathies (SpA) are a group of inflammatory disorders characterized by involvement of the spine and peripheral joints. In addition, entheseal inflammation is central to its pathogenesis. Adult axial SpA is seen in adults who present with inflammatory back pain (IBP) and may or may not have radiological evidence of sacroiliitis (1). Ankylosing spondylitis (AS) is the prototype of axial SpA that has a very strong association with human leukocyte antigen (HLA)-B27 with more than 95% patients being positive for it (2, 3). HLA-B27 along with environmental factors like mechanical stress and gut microbes are implicated in the pathogenesis of SpA.

Subclinical gut inflammation has been reported in 60% of patients with AS (4). Gastrointestinal symptoms and antibodies associated with gastrointestinal diseases, and IgA levels were associated with high disease activity in patients with SpA (5). Gut dysbiosis leads to leakage and translocation of bacterial products into the systemic circulation (6). These can then act via toll like receptors (TLR) s especially 2 and 4 (7) and activate monocytes/macrophages leading to the production of pro-inflammatory cytokines as well as IL-23 which skews the immune response towards IL-17 production by T cells and innate immune cells. Patients with AS and ERA show high expression of TLR 2 and 4 on monocytes as well as produce higher pro-inflammatory cytokines compared to healthy controls (HC) (8–11). In addition, we have recently shown that endogenous ligands like myeloid related protein (MRP)8/14 and Tenascin-C (TNC) are elevated in sera from AS patients compared to HC and their levels correlate with activity of the disease (12, 13).

Enthesitis related arthritis (ERA) category of juvenile idiopathic arthritis (JIA) is characterized by lower limb arthritis and enthesitis and is usually seen in boys older than 6 years (14). HLA-B27 is present in 60–80% of these children (15). IBP, the hallmark of AS develops in a proportion of patients after 5–10 years and about one-third progress to AS in adulthood (16). ERA patients also show gut dysbiosis and presence of autoantibodies associated with inflammation of gastrointestinal mucosa like antibodies to tissue transglutaminase or anti-*Saccharomyces cerevisiae* antibodies (17). They have elevated fecal calprotectin, high pro-inflammatory cytokines (TNF and IL-6) in synovial fluid (SF) and TLR4 endogenous ligands (TNC and MRP8/14) in serum (18–22). In addition, they also have increased TLR2 and 4 expression on monocytes in blood and SF (8).

Though ERA and AS show similarities in their clinical features, HLA-B27 association and some of the immune abnormalities, there are differences like higher prevalence of enthesitis and arthritis in juvenile onset SpA and a higher prevalence of IBP and uveitis in AS (23, 24) Thus, we aimed to see if the monocyte response seen in these two diseases is similar or different. To study this, we analyzed the frequency of cytokine producing monocytes in peripheral blood (PB) and synovial fluid mononuclear cells (SFMC) at baseline as well as on stimulation with both exogenous and endogenous TLR ligands. In addition, we also measured the production of cytokines as well as endogenous TLR ligands at baseline as well as on stimulation. Finally, we assessed the mRNA expression levels post-stimulation with the endogenous and exogenous TLR ligands.

## Patients and methods

### Patients

Since SpA is a male-dominant disease, and also to avoid gender difference as a potential confounder, we enrolled only male subjects. Adult axial SpA patients fulfilling the Assessment of SpondyloArthritis international Society [ASAS] classification criteria for axial SpA (25) and ERA patients fulfilling the JIA-International League of Associations for Rheumatology (ILAR) classification criteria (26) were included as study subjects. Young adult males were included as HC. None of the patient’s with ERA had psoriasis or inflammatory bowel disease.

Clinical assessments included Bath Ankylosing Spondylitis Disease Activity Index (BASDAI) (27) for adult axial SpA patients and Juvenile Spondyloarthropathy Disease Activity Score (JSpADA) (28) for ERA patients. These were assessed by the treating rheumatologist at the time of sample collection.

The PB was collected from all subjects whereas SF was collected from only those patients who required intra-articular steroid injection as a part of their treatment. All experiments were performed on whole blood (WB) as it is difficult to get a higher quantity of blood from children.

### Assessment of frequency of pro-inflammatory cytokine producing monocytes

500 μl PB was diluted (1:1) with RPMI (Sigma Aldrich, MO, USA) supplemented with 10% fetal bovine serum (FBS; Gibco, MA, USA) and 1% antibiotic (Antibiotic-antimycotic, Gibco, MA, USA) (complete culture medium) and cultured in 24-well plates for 4 h in a 5% CO_2_ incubator at 37 °C.

#### Stimulation of cells with TLR ligands

Cells were stimulated with following ligands for 4 h: LPS (100 ng/ml; a TLR4 ligand, Sigma Aldrich, MO, USA), Peptidoglycan (PG, 5 μg/ml; a TLR2 ligand, Sigma Aldrich, MO, USA), MRP8 (5 μg/ml; Abcam, Cambridge, UK) or TNC (10 μg/ml; Merck Millipore, MA, USA). Unstimulated cells served as baseline. 10 μg/ml Brefeldin A (Sigma Aldrich, MO, USA) was added as secretion inhibitor in all cultures.

#### Staining of cells and flow cytometry

Cells were surface-stained with fluorescent monoclonal antibody to CD14 (BD Biosciences, CA, USA). After RBC lysis (BD FACS lysis solution, BD Biosciences, CA, USA), cells were fixed and permeabilized (with BD Cytofix/Cytoperm solution, BD Biosciences, CA, USA). The cells were later stained for intracellular IL-6 and TNF, using fluorescent monoclonal antibodies (BD Biosciences, CA, USA). 10^5^ cells were acquired in the flow cytometer (Beckman Coulter, CA USA). The monocytes were gated in the side scatter (SSC) vs CD14 plot. In the CD14^+^ gate, cells were analysed and the frequency of CD14^+^ IL-6^+^ and CD14^+^ TNF^+^ cells was calculated using Navios software (Beckman Coulter, CA, USA). Gating strategy is shown in Additional file 1.

### In vitro production of pro-inflammatory cytokines and MMP3

500 μl PB was diluted (1:1) with complete culture medium and dispensed in 24-well plates. Cells were stimulated with following ligands (in a 5% CO_2_ incubator at 37 °C) for 24 h: LPS (2.5 μg/ml), PG (5 μg/ml), MRP8 (5 μg/ml), TNC (10 μg/ml). Unstimulated cells served as the baseline. In the culture supernatants, levels of TNF (BD OptEIA Kit, CA, USA), IL-6 (BD OptEIA Kit, CA, USA) and MMP3 (R&D systems, MN, USA) were measured by enzyme linked immunosorbent assay (ELISA), as per the manufacturer’s instructions. The minimum detection limit was 7.8 pg/ml for TNF, 4.7 pg/ml for IL-6 and 31.3 pg/ml for MMP3.

### Validation of pro-inflammatory cytokine production by quantitative PCR

In a subset of patients and HC (*n* = 5), 500 μl PB was diluted (1:1) with complete culture medium and dispensed in 24-well plates. Cells were stimulated with following ligands (in a 5% CO_2_ incubator at 37 °C) for 4 h: LPS (2.5 μg/ml), MRP8 (5 μg/ml) and TNC (10 μg/ml). Unstimulated cells served as baseline. The cells were later stored in TRIzol at − 80 °C (Thermo Fischer Scientific, MA, USA) till RNA isolation.

After thawing the cells and vortexing to facilitate lysis, the sample was centrifuged at 10,000×g for 10 mins at 4 °C. The supernatant was carefully aspirated and transferred to fresh microcentrifuge tubes. Following this, 200 μl of chloroform (Sigma Aldrich, MO, USA) was added to the suspension, mixed manually for 15 mins and kept at room temperature for 10 mins. It was then centrifuged at 12,000×g for 15 min at 4 °C. The aqueous layer was carefully aspirated and transferred to fresh microcentrifuge tubes. 500 μl of isopropyl-alcohol (Sigma Aldrich, MO, USA) was added to the separated aqueous layer and mixed. The suspension was centrifuged at 12,000×g for 10 mins at 4 °C. The RNA pellet was then washed twice with 1 ml of 75% ethanol (Merck Millipore, MA, USA) and centrifuged at 7500×g. The pellet was then dried at RT. Finally, the RNA pellet was dissolved in 10 μl of RNase free water. The absorbance was taken at 260 and 280 nm in Nanodrop spectrophotometer (Thermo Fisher, MA, USA).

cDNA was prepared using High-Capacity cDNA Reverse Transcription Kit (Applied Biosystems, CA, USA) as done in previous studies [6]. Subsequently, real-time PCR was performed using TaqMan Fast Advanced Master mix (Thermo Fischer, MA, USA). Taqman gene expression assay kits were purchased from Applied Biosystems (CA, USA), the IDs being Hs00174128_m1 (TNF), Hs00174131_m1 (IL-6) and HS02786624_g1 (GAPDH). Each 20 μl reaction mixture comprised of 10 μl TaqMan Fast Advanced Master mix, 1 μl TaqMan assay probe, 7 μl RNase free water and 2 μl cDNA. The reaction conditions in the real-time PCR amplification and detection instrument (LightCycler 480 Instrument II, Roche Molecular Systems Inc., CA, USA) were 50 °C for 2 min, 95 °C for 2 min and finally 40 PCR cycles of 95 °C for 3 s and 60 °C for 30 s.

GAPDH was used as a housekeeping gene. Relative fold change was determined by the ΔΔCt method (Ct = cycle threshold), where fold change =2^-ΔΔCt^ and ΔΔCt = [Ct_(TNF/IL-6)_-Ct_GAPDH_] for stimulated (LPS/TNC/MRP8) sample - [Ct_(TNF/IL-6)_-Ct_GAPDH_] for unstimulated sample. More than a 2-fold increase in expression was considered significant.

### In vitro production of TNC and MRP8/14 in response to LPS

500 μl PB was diluted (1:1) with complete culture medium and dispensed in 24 well plates. The cells were stimulated (24 h in a 5% CO_2_ incubator at 37 °C) with either LPS (2.5 μg/ml) or left unstimulated (baseline). Following this, levels of MRP8/14 (LEGEND MAX Human MRP8/14 Calprotectin ELISA kit, BioLegend, CA, USA) and TNC [Tenascin C large (FNIIIC), IBL International; Hamburg, Germany] in culture supernatants were measured by ELISA as per the manufacturer’s instructions. The minimum detection limit of MRP8/14 was 3.13 ng/ml and that of TNC was 0.38 ng/ml.

### Studies on synovial fluid cells

The SFMC’s were isolated by density gradient centrifugation using Histopaque 1077 (Sigma, MO, USA). Inflammatory response of SFMC was studied using the protocol described above for PB, except that 10^6^ SFMC/ml were used. Cells were stimulated with LPS, PG, TNC, MRP8 or left unstimulated (baseline); following which the TNF and IL-6 producing monocyte frequencies were measured as per the above-mentioned protocol for flow cytometry.

### Statistical analysis

All results are represented as median [Interquartile range (IQR)]. Intergroup comparison was done using non-parametric tests. Correlation with disease activity was assessed using Spearman’s correlation and values are expressed as r (95% confidence intervals). *p-*value < 0.05 was taken as significant. Exact *p* values are given unless it exceeded *p* < 0.0001. Graph pad prism 7 (trial version) was used for all statistical analysis.

## Results

### Patients

The study included 50 adult axial SpA and 52 ERA patients. In addition, 25 healthy adults [HC, median age 31, range 26–33 years] were included as controls. The demographic data and disease activity of patients was assessed at the time of collection of blood sample and is given in Table 1.

**Table 1:** Clinical and demographic details of adult axial SpA and ERA patients

Parameters	Axial SpA (*n* = 50)	ERA (*n* = 52)
Median age (range: in years)	29 (19–51)	15.5 (8–18)
Median disease duration (in years)	10 (4–23)	2.2 (0.3–10)
Clinical features (number)		
Active arthritis	34	50
Enthesitis	23	49
Uveitis	4	0
Sacroiliitis	50	10
Dactylitis	3	0
Joints involved		
Shoulder	6	5
Elbow	2	5
Wrist	6	9
Hip	10	10
Knee	15	34
Ankle	5	33
Subtalar	1	5
ESR [median (range)]	40 (2–133)	42 (4–135)
Drug usage (number)		
NSAIDs	31	32
Prednisolone	7	4
Methotrexate	13	4
HLA-B27 positive patients	47	42
Median BASDAI (IQR) (baseline)	4.5 (2.8)	
Median JSpADA (IQR) (baseline)		4.5 (1.75)

Note: The drug usage as well as BASDAI and JSpADA were recorded at the time of collection of the sample. *SpA* Spondyloarthropathy, *ERA* enthesitis related arthritis, *BASDAI* (Bath Ankylosing Spondylitis Disease Activity Index), *JSpADA* (Juvenile Spondyloarthropathy Disease Activity Score) are represented as median (IQR)

Whole blood diluted 1:1 with complete culture medium was used for assessing the pro-inflammatory cytokine producing monocyte frequency, production of pro-inflammatory cytokines, MMP3 and TLR4 endogenous ligands as well as the expression of pro-inflammatory cytokine producing gene.

### Frequency of pro-inflammatory cytokine producing monocytes in blood

#### TNF producing monocyte frequency

**Fig. 1:**
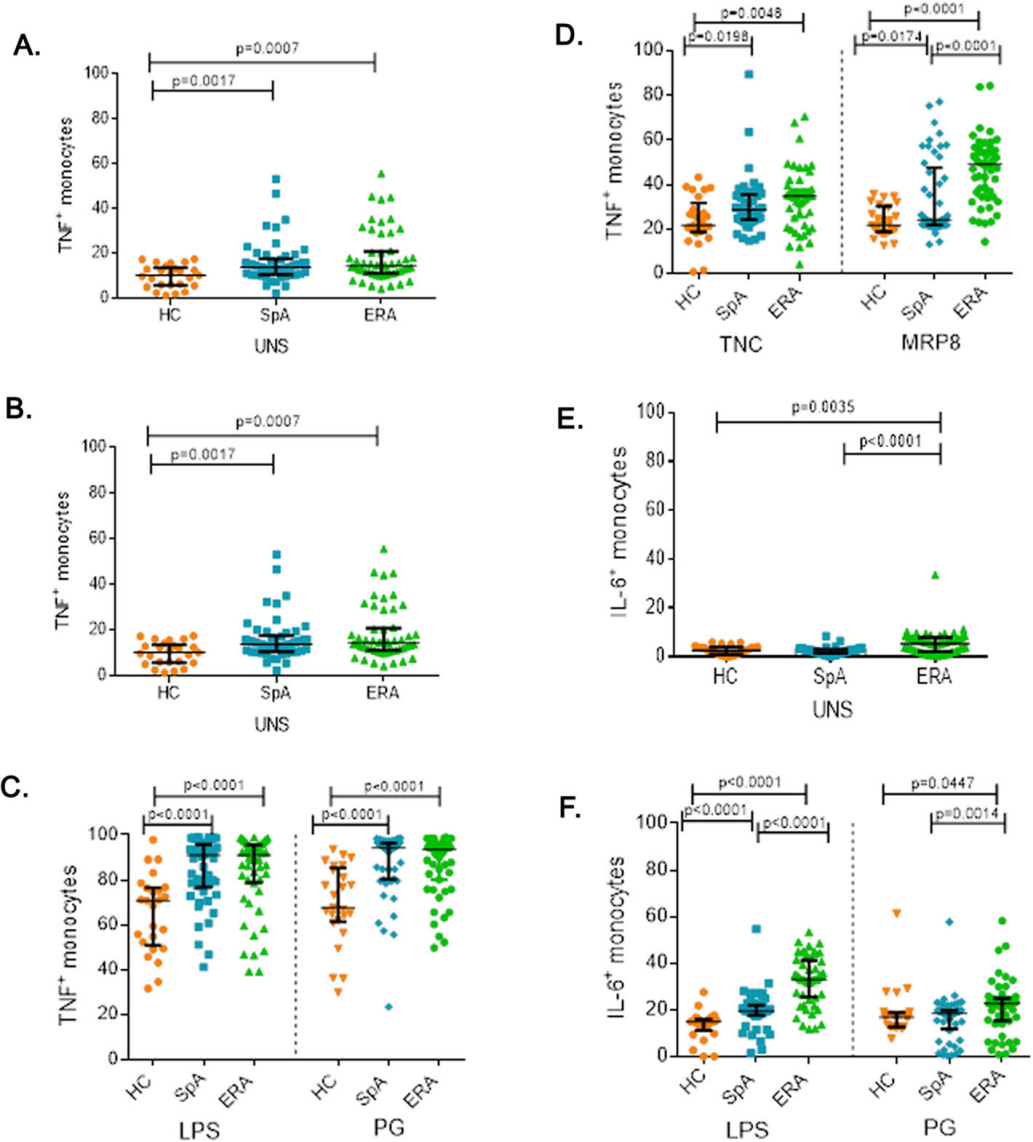
TNF and IL-6 producing monocyte frequency (PB) in HC, SpA and ERA patients. The figure shows scatter plots representing frequency of TNF and IL-6 producing monocytes in PB in HC (25), SpA (50) and ERA (52) patients as analysed via flow cytometry. Each dot represents an individual sample. Horizontal line represents median. WB diluted 1:1 with complete culture medium was used for assessing the pro-inflammatory cytokine producing monocyte frequency. Frequency of TNF producing monocytes in response to (**a**). No stimulation (**b**). LPS or PG stimulation (**c**). TNC or MRP8 stimulation. Frequency of IL-6 producing monocytes in response to (**d**). No stimulation. (**e**.) LPS or PG stimulation (**f**). TNC or MRP8 stimulation. *WB:* whole blood, *HC:* healthy controls, *SpA:* Spondyloarthropathy, *ERA:* enthesitis related arthritis, *Uns*- unstimulated, *LPS-* Lipopolysaccaride, *PG-* peptidoglycan, *TNC-* Tenascin-C and *MRP8-*Myeloid related protein 8, *TNF:* tumor necrosis factor, *IL-6:* Interleukin-6

Adult axial SpA and ERA patients had similar frequency of TNF producing monocytes at baseline which were higher than HC (Fig. 1a). Post-stimulation with exogenous (LPS and PG) or endogenous (TNC) TLR ligands also, both patient groups had similar frequency of TNF producing monocytes which was higher than HC (*p* < 0.0001). However, MRP8 stimulation, led to higher TNF producing monocyte frequency in ERA patients compared to adult axial SpA (*p* < 0.0001) (Fig. 1b, c, Additional file 2).

#### IL-6 producing monocyte frequency

At baseline as well as post stimulation with exogenous TLR ligands (LPS and PG), ERA patients had higher IL-6 producing monocyte frequency compared to adult axial SpA (Fig. 1d, e). However, stimulation with endogenous ligands of TLR4 (TNC and MRP8) led to similar IL-6 producing monocyte frequency in both adult axial SpA and ERA patients which was observed to be higher than HC (*p* < 0.0001; Fig. 1f) (Additional file 2).

#### Correlation with disease activity

**Fig. 2:**
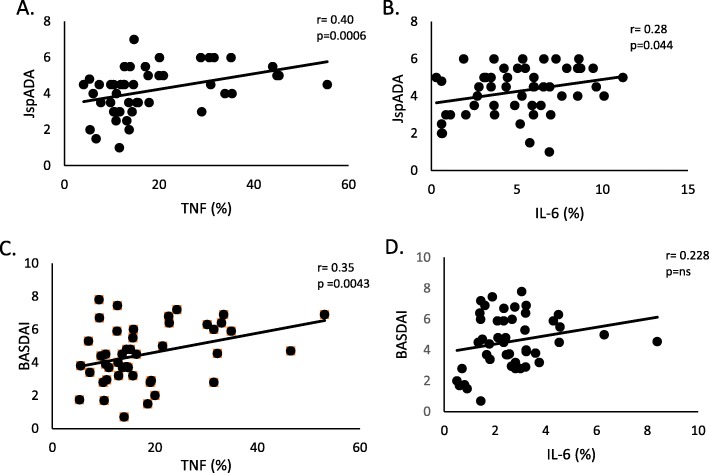
Baseline pro-inflammatory monocyte frequency correlation (PB) with disease activity scores in SpA and ERA patients. Correlation in ERA patients: **a**. baseline TNF% vs JSpADA **b**. baseline IL-6% vs JspADA. Correlation in SpA patients: **c**. baseline TNF% vs BASDAI, **d**. baseline IL-6% vs BASDAI. *PB:* peripheral blood, *BASDAI* Bath Ankylosing Spondylitis Disease Activity Index, *JspADA* Juvenile spondyloarthritis Disease Activity Score

The baseline frequency of TNF producing monocytes from ERA and adult axial SpA patients showed correlation with their corresponding disease activity scores [ERA- JSpADA (*r* = 0.40; *p* = 0.0006); adult axial SpA- BASDAI (*r* = 0.35, *p* = 0.0043)] (Fig. 2a, c).

Baseline IL-6 producing monocyte frequency from ERA patients showed a modest correlation with JSpADA (*r* = 0.28, *p* = 0.044) (Fig. 2b).

### Pro-inflammatory cytokine and MMP3 production in response to TLR ligands

**Fig. 3:**
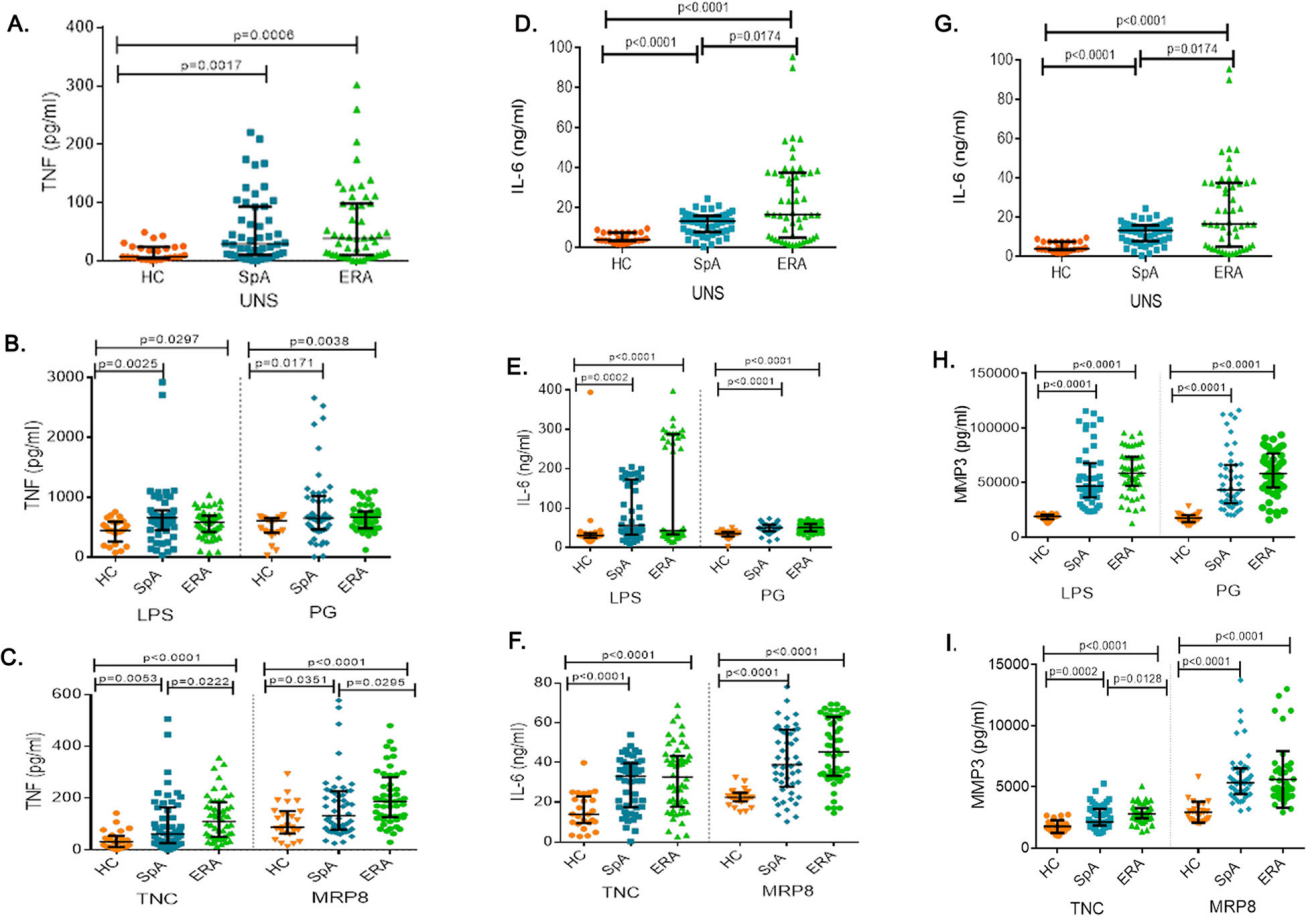
TNF, IL-6 and MMP3 production (PB) in HC, SpA and ERA patients**.** Scatter plots representing TNF, IL-6 and MMP3 production in PB in three group of subjects, HC (25), SpA (50) and ERA (52) patients as measured via ELISA. Each dot represents an individual sample. Horizontal line represents median. WB diluted 1:1 with complete culture medium was used for assessing the pro-inflammatory cytokine production post stimulation with endogenous and exogenous ligands. TNF production in response to (**a**). No stimulation (**b**). LPS or PG stimulation (**c**). TNC or MRP8 stimulation. IL-6 production in response to (**d**). No stimulation. (**e**.) LPS or PG stimulation (**f**). TNC or MRP8 stimulation. MMP3 production in response to (**g**). No stimulation. (**h**.) LPS or PG stimulation (**i**). TNC or MRP8 stimulation. *WB:* whole blood, *HC:* healthy controls, *SpA:* Spondyloarthropathy, *ERA:* enthesitis related arthritis, *Uns*- unstimulated, *LPS-* Lipopolysaccaride, *PG-* peptidoglycan, *TNC-* Tenascin-C and *MRP8-*Myeloid related protein 8, *TNF:* tumor necrosis factor, *IL-6:* Interleukin-6, *MMP3:* Matrix metalloproteinase 3

At baseline, as well as post stimulation with TLR exogenous ligands (LPS and PG), adult axial SpA and ERA patients showed similar production of TNF which was higher than HC (Fig. 3a, b). Stimulation with the endogenous ligands of TLR4 however, led to higher production of TNF from ERA patients compared to adult axial SpA (Fig. 3c) (Additional file 3).

At baseline, IL-6 production was higher in ERA patients than adult axial SpA (*p* = 0.0174; Fig. 3d). However, stimulation with exogenous (LPS and PG) as well as endogenous (TNC and MRP8) TLR ligands led to similar production of IL-6 from adult axial SpA and ERA patients which was higher than HC (Fig. 3e, f; Additional file 3).

At baseline, the MMP3 production level was observed to be higher in ERA patients compared to adult axial SpA (*p* = 0.0164; Fig. 3g). However, stimulation with LPS, PG and MRP8 led to similar MMP3 production from adult axial SpA and ERA patients which was higher than HC. On stimulation with TNC however, ERA patients produced more MMP3 as compared to adult axial SpA patients (*p* = 0.012) (Fig. 3h, i; Additional file 3).

### Validation of pro-inflammatory cytokine production by quantitative PCR

Comparison of the normalized Ct values indicate that stimulation with exogenous (LPS) as well as endogenous (TNC and MRP8) TLR ligands showed similar TNF fold change values in ERA and adult axial SpA patients which was higher than HC (*p* = 0.0079). Similar results were also observed for IL-6 (*p* = 0.0079) (Additional file 4).

### Production of endogenous ligands in response to LPS

**Fig. 4:**
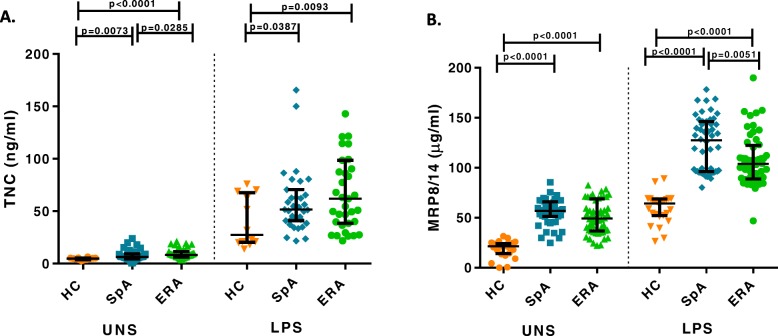
TNC and MRP8/14 production (PB) in HC, adult axial SpA and ERA patients. Scatter plots representing TNC and MRP8/14 production in PB in three group of subjects, as measured via ELISA. Each dot represents an individual sample. Horizontal line represents median. WB diluted 1:1 with complete culture medium was used for assessing the endogenous ligand production post stimulation with LPS. **a**. TNC production from HC (12), adult axial SpA (36) and ERA (36) patients in response to no stimulation or LPS stimulation **b**. MRP8/14 production in HC (25), adult axial SpA (50) and ERA (52) patients in response to no stimulation or LPS stimulation. *WB:* whole blood, *HC:* healthy controls, *SpA:* Spondyloarthropathy, *ERA:* enthesitis related arthritis, *Uns*- unstimulated, *LPS-* Lipopolysaccaride, *TNC-* Tenascin-C and *MRP8/14-*Myeloid related protein 8/14

In unstimulated cultures, the TNC production was higher in ERA patients compared to adult axial SpA patients (*p* = 0.0285). However, on stimulation with LPS, the levels were observed to be similar in adult axial SpA and ERA patients which was higher than HC (Fig. 4a; Additional file 5).

At baseline, adult axial SpA and ERA patients showed similar production of MRP8/14 which was higher than HC (*p* < 0.0001). However, post stimulation with LPS, the MRP8/14 production was observed to be higher in adult axial SpA patients compared to ERA (*p* = 0.0051) (Fig. 4b; Additional file 5).

### Frequency of pro-inflammatory cytokine producing monocytes in synovial fluid

**Fig. 5:**
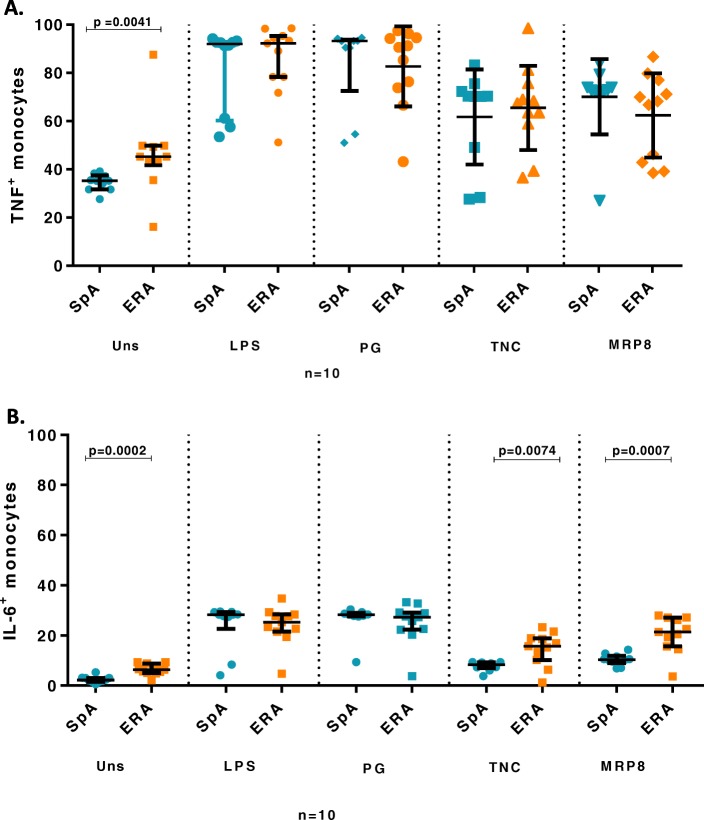
TNF and IL-6 producing monocytes in SFMC in SpA vs ERA patients. Scatter plots representing frequency of TNF and IL-6 producing monocytes in SFMC in SpA (10) and ERA (10) patients in response to exogenous and endogenous TLR4 ligands analysed via flow cytometry. Each dot represents an individual sample. Horizontal line represents median. 10^6^ SFMC/ml in complete culture medium was used. **a**. Frequency of TNF producing monocytes (**b**). Frequency of IL-6 producing monocytes. *WB:* whole blood, *SpA:* Spondyloarthropathy, *ERA:* enthesitis related arthritis *Uns*- unstimulated, *LPS-* Lipopolysaccaride, *PG-* peptidoglycan, *TNC-* Tenascin-C and *MRP8-*Myeloid related protein 8, *TNF:* tumor necrosis factor, *IL-6:* Interleukin-6

10^6^ SFMC/ml in complete culture medium was used for assessing the pro-inflammatory cytokine producing monocytes in SF. The SF samples were available from 10 adult axial SpA and 10 ERA patients. At baseline, the TNF producing monocyte frequency was observed to be higher in ERA patients compared to adult axial SpA (*p* = 0.0041). However, stimulation with the endogenous as well as exogenous ligands of TLR4 led to similar TNF producing monocyte frequency from both patient subsets (Fig. 5a) (Additional file 6).

At baseline as well as post stimulation with the endogenous ligands (TNC and MRP8/14), IL-6 producing monocyte frequency was elevated in ERA patients compared to adult axial SpA patients. Stimulation with the exogenous TLR ligands led to similar frequency of IL-6 producing monocytes from both patient subsets. (Fig. 5b) (Additional file 6).

## Discussion

Both ERA and adult axial SpA patients had similar monocyte responses with minor differences. Both had higher frequencies of pro inflammatory cytokine producing monocytes as well as TNF, IL-6, MMP3, TNC and MRP8/14 production at baseline and post-stimulation with TLR exogenous and endogenous ligands as compared to HC. The cytokine producing monocyte frequency at baseline also correlated with the disease activity. Stimulation with TLR ligands led to similar increase in frequency of TNF producing monocytes in SFMC’s. However, the IL-6 producing monocyte frequency was higher in ERA patients than adult axial SpA post stimulation with exogenous ligands of TLR4.

Adult axial SpA and ERA patients had higher frequency of cytokine producing monocyte frequency at baseline as compared to HC. This suggests towards the presence of pre-activated monocytes in both patient subsets. Previously in patients with adult axial SpA, elevated frequencies of pro-inflammatory cytokine producing monocytes was observed. However, this was observed only in patients receiving conventional disease modifying anti-rheumatic drugs (DMARDs) and not in those receiving anti-TNF therapy (29). Methotrexate (MTX) has not been observed to inhibit cytokine production by monocytes (30) while prednisolone has been shown to reduce TNF production from monocytes (31). None of the patients included in the present study were receiving anti-TNF therapy and a small proportion of them were on MTX and prednisolone.

Positive correlation of the baseline pro-inflammatory cytokine producing monocyte frequency with disease activity was observed for both adult axial SpA and ERA patients. This suggests that cytokines produced by monocytes contribute to inflammation. An association between the serum levels of inflammatory mediators and disease activity in AS has been reported (32, 33) Moreover, therapeutic success of anti-TNF agents in both AS and ERA further supports the role of pro-inflammatory cytokines in SpA (34, 35).

The elevated production of IL-6 as well as MMP3 from ERA patients at baseline as compared to adult axial SpA may indicate that the monocytes from ERA patients are more activated and this could be related to higher disease activity as reflected by presence of more active peripheral arthritis and enthesitis in ERA patients as compared to adult axial SpA. The real reason behind the difference in IL-6 production compared to IL-6 producing monocyte frequency in the patient subsets is difficult to explain. However, some other blood cells along with monocytes may have contributed to IL-6 production in WB cultures whereas in flow cytometry, we have assessed only the IL-6 producing monocyte frequency.

The present study also provides the data that endogenous ligands like MRP8/14 and TNC also activate monocytes in patients with adult axial SpA and ERA. Here again, monocytes from ERA patients showed a slightly higher response as compared to adult axial SpA. MRP8/14 has been observed to be expressed in monocytes and infiltrating neutrophils in the inflamed joints of JIA patients (36). Stimulation of PBMCs from healthy subjects with MRP8/14 has already been shown to cause production of pro-inflammatory cytokines (37). TNC promotes the recruitment of monocytes/macrophages to the site of inflammation (38). It also binds to TLR4 on monocyte/macrophages and causes production of pro-inflammatory cytokines in a dose dependent manner (39). These cytokines can induce production of TNC through an ‘auto amplification’ loop (40).

Endogenous ligands were less potent then LPS in stimulating monocytes. TNC has also been shown to elicit less potent cytokine response than LPS in macrophages (39). It is known that endogenous ligands like TNC enhance expression of extracellular matrix proteins which work towards tissue repair (41), whereas LPS activates several downstream signalling pathways, causing production of pro-inflammatory cytokines (42).

Increased production of MMP3 on stimulation with endogenous ligands suggests that MMP3 production could be another mechanism by which the activated monocytes cause joint damage. MMP3 is a potent protease which causes degradation of matrix as well as cartilage, leading to joint damage. Children suffering from ERA have increased levels of MMP3 in the serum (43).

We have shown that endogenous ligands induce pro-inflammatory response from monocytes in patients and it has been reported that both MRP8/14 and TNC levels are elevated in patients with ERA and AS (12, 13, 18, 21). We studied the production of these ligands on stimulation of monocytes and found that indeed, patients cells produced higher levels of TNC & MRP8/14 as compared to HC. MRP8/14 and TNC are produced by monocytes upon stimulation by microbes or stress (44, 45).

SFMC’s from both adult axial SpA and ERA patients had similar frequency of TNF producing monocytes after stimulation. However, the presence of higher IL-6 producing monocyte frequency from ERA patients than adult axial SpA post stimulation with endogenous ligands suggests that at the local site, the monocyte/macrophage lineage cells are more activated in ERA patients. This could be due to selective homing of activated cells in the synovium or their higher activation at local site due to excessive production of endogenous ligands. Indeed, levels of MRP8/14 in SF are higher than plasma in children with ERA (21).

The strength of this study includes a good sample size, validation of data using three methods, i.e., frequency of cytokine producing monocytes, cytokine levels in culture supernatants and mRNA quantification, and inclusion of SF samples. The limitations of this study are lack of healthy children control, significant disease duration at time of inclusion, use of glucocorticoids and MTX by a proportion of patients at the time of analysis. However, the abnormalities seen are similar in juvenile and adult SpA, suggesting that they are not influenced by the age of patients but are specific to the disease. Though glucocorticoids and MTX can affect cytokine production however, we did not find any difference in the two groups (data not shown). It would be ideal to study cytokine production by monocytes separated from WB. However, this requires a minimum of 15–20 ml blood to get 1.5–2 million monocytes which is difficult in children. Thus, monocyte response is mostly similar in ERA and adult axial SpA.

## Conclusion

All this data suggests that ERA and adult axial SpA share a common pathogenesis and innate cells like monocytes play an important role by producing pro-inflammatory cytokines as well as endogenous TLR ligands and thus setting a self-perpetuating cycle of inflammation, the so called ‘auto-inflammation’. Therapies targeting TNC have shown positive results in immune inflammatory disease (46). This study is in line with the growing belief that differences between paediatric and adult-onset rheumatic diseases are minimal (47). This may also suggest that these two diseases are a continuum and may explain progression of one-third of patients with ERA into adult axial SpA later (16).

## Supplementary information

**Additional file 1.** Gating strategy for flow cytometry analysis. Demonstration of gating strategy for flow cytometric analysis of CD14 monocytes from PB and SFMC samples. In this example, 500 μl PB (from HC) + 500 μl cRPMI was left unstimulated for 4 h with brefildin A. the cells were then surface stained with CD14 (FITC) monoclonal antibody and IL-6 (APC) and TNF (PE) intracellular monoclonal antibodies. The CD14^+^ monocytes were gated on the SSC vs CD14-FITC plot. The monocytes were then analysed for CD14^+^TNF^+^ cells and CD14^+^IL-6^+^ cells. The gating was done on the basis of lymphocyte exclusion method of flow cytometry analysis.

**Additional file 2.** TNF^+^ and IL-6^+^ monocytes after stimulation with TLR ligands (LPS, PG, TNC and MRP8) in PB in patients and HC. Table showing the frequency of TNF and IL-6 producing monocytes on stimulation with endogenous (LPS and TNC) and exogenous (TNC and MRP8) TLR ligands in SpA, ERA patients and HC. WB diluted 1:1 with complete culture medium was used.

**Additional file 3.** MMP3, TNF and IL-6 production after stimulation with TLR ligands (LPS, PG, TNC and MRP8) in patients and HC. Table showing the level of TNF, IL-6 and MMP3 production on stimulation with endogenous (LPS and TNC) and exogenous (TNC and MRP8) TLR ligands in SpA, ERA patients and HC. WB diluted 1:1 with complete culture medium was used.

**Additional file 4 **TNF and IL-6 mRNA fold change (PB) in HC, SpA and ERA patients. Scatter plots representing TNF and IL-6 mRNA fold change in PB in three group of subjects, HC (5), SpA (5) and ERA (5) patients as measured via quantitative PCR Each dot represents an individual sample. Horizontal line represents mean. Fold change =2^-ΔΔCt^ and ΔΔCt = [Ct_(TNF/IL-6)_-Ct_GAPDH_] stimulated sample (LPS/TNC/MRP8) - [Ct_(TNF/IL-6)_-Ct_GAPDH_ unstimulated sample. TNF mRNA fold change in response to (A). LPS stimulation (B). TNC stimulation (C). MRP8 stimulation. IL-6 mRNA fold change in response to (D). LPS stimulation (E). TNC stimulation (F). MRP8 stimulation. WB diluted 1:1 with complete culture medium was used. *HC:* healthy controls, *SpA:* Spondyloarthropathy, *ERA:* enthesitis related arthritis, *Uns*- unstimulated, *LPS-* Lipopolysaccaride, *PG-* peptidoglycan, *TNC-* Tenascin-C and *MRP8-*Myeloid related protein 8, *TNF:* tumor necrosis factor, *IL-6:* Interleukin-6.

**Additional file 5.** TNC and MRP8/14 production after stimulation with LPS in patients and HC. Table showing the level of TNC and MRP8 production on stimulation with endogenous (LPS) TLR4 ligand in SpA, ERA patients and HC. WB diluted 1:1 with complete culture medium was used.

**Additional file 6.** TNF^+^ and IL-6^+^ monocytes after stimulation with TLR ligands (LPS, PG, TNC and MRP8) in SFMC in SpA and ERA patients. Table showing the frequency of TNF and IL-6 producing monocytes on stimulation with endogenous (LPS and TNC) and exogenous (TNC and MRP8) TLR ligands in SpA and ERA patients. 10^6^ SFMC/ml in complete culture medium was used.

## Data Availability

The datasets used and analysed during the current study are available from the corresponding author on reasonable request.

## Abbreviations

AS: Ankylosing Spondylitis
ASAS: Assessment of SpondyloArthritis international Society
BASDAI: Bath Ankylosing Spondylitis Disease Activity Index
cDNA: Complementary DNA
DMARDs: Disease modifying anti-rheumatic drugs
ELISA: Enzyme linked immunosorbent assay
ERA: Enthesitis Related Arthritis
GAPDH: Glyceraldehyde dehydrogenase
HC: Healthy Control HLA-B27 Human Leukocyte Antigen-B27
IBP: Inflammatory back pain
IL-17: Interleukin-17
IL-23: Interleukin-23
ILAR: International League of Associations for Rheumatology IL-6 Interleukin-6
JIA: Juvenile idiopathic arthritis
JSpADA: Juvenile Spondyloarthropathy Disease Activity Score LPS Lipopolysaccharide
MMP3: Matrix metalloproteinase 3
MRP: Myeloid Related Protein
MTX: Methotrexate
PB: Peripheral Blood
PCR: Polymerase chain reaction PG Peptidoglycan
RBC: Red blood cells
RNA: Ribonucleic acid
RPMI: Roswell park memorial institute
SF: Synovial Fluid
SFMC: Synovial Fluid Mononuclear Cells
SpA: Spondyloarthropathy
SSC: Side scatter
TLR: Toll Like Receptor
TNC: Tenascin-C
TNF: Tumour necrosis factor
WB: Whole blood
