# Supplementary material for: Patients with enthesitis related arthritis show similar monocyte function pattern as seen in adult axial spondyloarthropathy
Source: Pediatr Rheumatol Online J. 2020 Jan 15;18:6. doi: 10.1186/s12969-020-0403-9 (PMC6964050; doi:10.1186/s12969-020-0403-9)
Supplement: Supplementary file 1 — Additional file 1. Gating strategy for flow cytometry analysis. Demonstration of gating strategy for flow cytometric analysis of CD14 monocytes from PB and SFMC samples. In this example, 500 μl PB (from HC) + 500 μl cRPMI was left unstimulated for 4 h with brefildin A. the cells were then surface stained with CD14 (FITC) monoclonal antibody and IL-6 (APC) and TNF (PE) intracellular monoclonal antibodies. The CD14+ monocytes were gated on the SSC vs CD14-FITC plot. The monocytes were then analysed for CD14+TNF+ cells and CD14+IL-6+ cells. The gating was done on the basis of lymphocyte exclusion method of flow cytometry analysis. [file 12969_2020_403_MOESM1_ESM.docx]

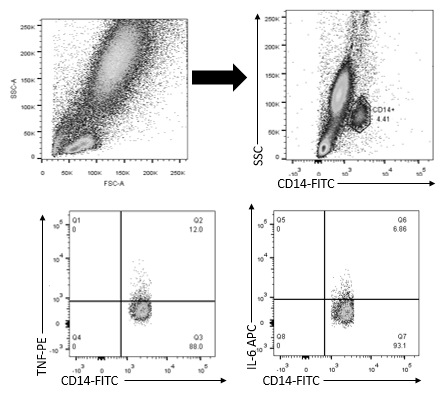


**Additional file 1: Gating strategy for flow cytometry analysis**. Demonstration of gating strategy for flow cytometric analysis of CD14 monocytes from PB and SFMC samples. In this example, 500 μl PB (from HC) + 500 μl cRPMI was left unstimulated for 4 hours with brefildin A. the cells were then surface stained with CD14 (FITC) monoclonal antibody and IL-6 (APC and TNF (PE) intracellular monoclonal antibodies. The CD14+ monocytes were gated on the SSC vs CD14-FITC plot. The monocytes were then analysed for CD14+TNF+ cells and CD14+IL-6+ cells. The gating was done on the basis of lymphocyte exclusion method of flow cytometry analysis.
