# Supplementary material for: Patients with enthesitis related arthritis show similar monocyte function pattern as seen in adult axial spondyloarthropathy
Source: Pediatr Rheumatol Online J. 2020 Jan 15;18:6. doi: 10.1186/s12969-020-0403-9 (PMC6964050; doi:10.1186/s12969-020-0403-9)
Supplement: Supplementary file 2 — Additional file 2. TNF+ and IL-6+ monocytes after stimulation with TLR ligands (LPS, PG, TNC and MRP8) in PB in patients and HC. Table showing the frequency of TNF and IL-6 producing monocytes on stimulation with endogenous (LPS and TNC) and exogenous (TNC and MRP8) TLR ligands in SpA, ERA patients and HC. WB diluted 1:1 with complete culture medium was used. [file 12969_2020_403_MOESM2_ESM.docx]

**Additional file 2: TNF^+^ and IL-6^+^ monocytes after stimulation with TLR ligands (LPS, PG, TNC and MRP8) in PB in patients and HC.**

|  | HC (n=25) | SpA (n=50) | ERA (n=52) |
| --- | --- | --- | --- |
| TNF^+^ monocytes (%) | | | |
| *Unstimulated* | *10 (7.86)* | *13.65 (7)** | *14.1 (9.77)** |
| *LPS stimulation* | *70.8 (25.75)* | *90.9 (19.02)** | *90.9 (16.65)** |
| *PG stimulation* | *67.8 (23.95)* | *94.1 (16.02)** | *93.4 (16.5)** |
| *TNC stimulation* | *21.8 (13.1)* | *28.7 (11.2)** | *34.9 (12.15)** |
| *MRP8 stimulation* | *21.9 (11.55)* | *24.2 (25.75)** | *49.1 (20.25)** |
| IL-6^+^ monocytes (%) | | | |
| *Unstimulated* | *2.51 (2.99)* | *2.34 (1.427)* | *5.25 (5.84)** |
| *LPS stimulation* | *14.7 (4.85)* | *32.9 (15.82)** | *32.9 (15.82)** |
| *PG stimulation* | *16.8 (6.17)* | *18.35 (8.1)* | *22.7 (9.7)** |
| *TNC stimulation* | *8.56 (8.76)* | *10.7 (2.96)** | *11.83 (5.23)** |
| *MRP8 stimulation* | *11.7 (9.92)* | *16.3 (2.8)** | *19.8 (10.28)** |

Results are expressed as median (IQR), * p <0.05 compared to healthy controls (HC). Exact p values are given in figure 1. *SpA*: Spondyloarthropathy; *ERA*: Enthesitis related arthritis
