# Supplementary material for: Patients with enthesitis related arthritis show similar monocyte function pattern as seen in adult axial spondyloarthropathy
Source: Pediatr Rheumatol Online J. 2020 Jan 15;18:6. doi: 10.1186/s12969-020-0403-9 (PMC6964050; doi:10.1186/s12969-020-0403-9)
Supplement: Supplementary file 3 — Additional file 3. MMP3, TNF and IL-6 production after stimulation with TLR ligands (LPS, PG, TNC and MRP8) in patients and HC. Table showing the level of TNF, IL-6 and MMP3 production on stimulation with endogenous (LPS and TNC) and exogenous (TNC and MRP8) TLR ligands in SpA, ERA patients and HC. WB diluted 1:1 with complete culture medium was used. [file 12969_2020_403_MOESM3_ESM.docx]

**Additional file 3: MMP3, TNF and IL-6 production after stimulation with TLR ligands (LPS, PG, TNC and MRP8) in patients and HC.**

|  | HC (n=25) | SpA (n=50) | ERA (n=52) |
| --- | --- | --- | --- |
| TNF production (pg/ml) | | | |
| *Unstimulated* | *6.7(18.59)* | *28.89 (82.89)** | *38.60 (88.29) ** |
| *LPS stimulation* | *447.42 (331.81)* | *659.52 (321.99)** | *576.92 (273.41)** |
| *PG stimulation* | *606.13 (245.14)* | *654 (561.93)** | *666.99 (238.74)** |
| *TNC stimulation* | *31.17 (43.53)* | *44.48 (92)** | *75.488 (113.94)** |
| *MRP8 stimulation* | *87.76 (85.74)* | *138.76 (145.28)** | *167.34 (119.06)** |
| IL-6 production (ng/ml) | | | |
| *Unstimulated* | *3.6 (3.2)* | *13.12 (8.06)** | *16.49 (32.53)** |
| *LPS stimulation* | *30.17 (11.3)* | *41.01 (30.17)** | *35.89 (12.82)** |
| *PG stimulation* | *35.42 (7.6)* | *49.41 (16.49)** | *50.93 (18.76)** |
| *TNC stimulation* | *13.9 (13.49)* | *33.10 (22.24)** | *29.57 (24.03)** |
| *MRP8 stimulation* | *22.38 (4.88)* | *44.26 (29.17)** | *45.27 (29.69)** |
| MMP3 production (pg/ml) | | | |
| *Unstimulated* | *104 (25.5)* | *280(250)** | *35.5 (120.75)** |
| *LPS stimulation* | *18930 (3974.5)* | *46950 (31390)** | *60634 (20645.25)** |
| *PG stimulation* | *17530 (6272)* | *43470 (35230)** | *60573.5 (32587.25)** |
| *TNC stimulation* | *1683 (791.5)* | *2150 (1560)** | *2782 (780.5)** |
| *MRP8 production* | *2849 (1062)* | *5340 (2100)** | *4826.5 (1991)** |

Results are expressed as median (IQR), * p <0.05 compared to healthy controls (HC). Exact p values are given in figure 3. *SpA:* Spondyloarthropathy; *ERA:* Enthesitis related arthritis
