# Supplementary material for: Patients with enthesitis related arthritis show similar monocyte function pattern as seen in adult axial spondyloarthropathy
Source: Pediatr Rheumatol Online J. 2020 Jan 15;18:6. doi: 10.1186/s12969-020-0403-9 (PMC6964050; doi:10.1186/s12969-020-0403-9)
Supplement: Supplementary file 4 — Additional file 4 TNF and IL-6 mRNA fold change (PB) in HC, SpA and ERA patients. Scatter plots representing TNF and IL-6 mRNA fold change in PB in three group of subjects, HC (5), SpA (5) and ERA (5) patients as measured via quantitative PCR Each dot represents an individual sample. Horizontal line represents mean. Fold change =2-ΔΔCt and ΔΔCt = [Ct(TNF/IL-6)-CtGAPDH] stimulated sample (LPS/TNC/MRP8) - [Ct(TNF/IL-6)-CtGAPDH unstimulated sample. TNF mRNA fold change in response to (A). LPS stimulation (B). TNC stimulation (C). MRP8 stimulation. IL-6 mRNA fold change in response to (D). LPS stimulation (E). TNC stimulation (F). MRP8 stimulation. WB diluted 1:1 with complete culture medium was used. HC: healthy controls, SpA: Spondyloarthropathy, ERA: enthesitis related arthritis, Uns- unstimulated, LPS- Lipopolysaccaride, PG- peptidoglycan, TNC- Tenascin-C and MRP8-Myeloid related protein 8, TNF: tumor necrosis factor, IL-6: Interleukin-6. [file 12969_2020_403_MOESM4_ESM.docx]

**D.**

**A.**

**E.**

**B..**

**F.**

**C.**

**Additional file 4: TNF and IL-6 mRNA fold change (PB) in HC, SpA and ERA patients.**

Scatter plots representing TNF and IL-6 mRNA fold change in PB in three group of subjects, HC (5), SpA (5) and ERA (5) patients as measured via quantitative PCR Each dot represents an individual sample. Horizontal line represents mean. Fold change =2^-ΔΔCt^ and ΔΔCt = [Ct_(TNF/IL-6)_-Ct_GAPDH_] stimulated sample (LPS/TNC/MRP8) - [Ct_(TNF/IL-6)_-Ct_GAPDH_ unstimulated sample. TNF mRNA fold change in response to (A). LPS stimulation (B). TNC stimulation (C). MRP8 stimulation. IL-6 mRNA fold change in response to (D). LPS stimulation (E). TNC stimulation (F). MRP8 stimulation. WB diluted 1:1 with complete culture medium was used. *WB:* whole blood, *HC:* healthy controls, *SpA:* Spondyloarthropathy, *ERA:* enthesitis related arthritis, *Uns*- unstimulated, *LPS-* Lipopolysaccaride, *PG-* peptidoglycan, *TNC-* Tenascin-C and *MRP8-*Myeloid related protein 8, *TNF:* tumor necrosis factor, *IL-6:* Interleukin-6.
