# Supplementary material for: Patients with enthesitis related arthritis show similar monocyte function pattern as seen in adult axial spondyloarthropathy
Source: Pediatr Rheumatol Online J. 2020 Jan 15;18:6. doi: 10.1186/s12969-020-0403-9 (PMC6964050; doi:10.1186/s12969-020-0403-9)
Supplement: Supplementary file 5 — Additional file 5. TNC and MRP8/14 production after stimulation with LPS in patients and HC. Table showing the level of TNC and MRP8 production on stimulation with endogenous (LPS) TLR4 ligand in SpA, ERA patients and HC. WB diluted 1:1 with complete culture medium was used. [file 12969_2020_403_MOESM5_ESM.docx]

**Additional file 5:** **TNC and MRP8/14 production after stimulation with LPS in patients and HC**

|  | **HC** | **SpA** | **ERA** |
| --- | --- | --- | --- |
| **TNC** (ng/ml) | **(n=12)** | **(n=36)** | **(n=36)** |
| Unstimulated | *4.6 (1.5)* | *6 (4.8)** | *8.3 (5.1)** |
| LPS stimulation | *27.4 (47.4)* | *51.53 (29.7)** | *61.88 (60.14)** |
| **MRP8/14** (μg/ml) | **(n=25)** | **(n=50)** | **(n=52)** |
| Unstimulated | *21.45 (10.17)* | *56.99 (14.56)** | *48.28 (32.14)** |
| LPS stimulation | *64.29 (16.78)* | *127.37(50.19)** | *103.8(33.68)** |

Results are expressed as median (IQR), * p <0.05 as compared to healthy controls (HC). Exact p values are given in figure 4. *SpA:* Spondyloarthropathy; *ERA:* Enthesitis related arthritis
