# Supplementary material for: Patients with enthesitis related arthritis show similar monocyte function pattern as seen in adult axial spondyloarthropathy
Source: Pediatr Rheumatol Online J. 2020 Jan 15;18:6. doi: 10.1186/s12969-020-0403-9 (PMC6964050; doi:10.1186/s12969-020-0403-9)
Supplement: Supplementary file 6 — Additional file 6. TNF+ and IL-6+ monocytes after stimulation with TLR ligands (LPS, PG, TNC and MRP8) in SFMC in SpA and ERA patients. Table showing the frequency of TNF and IL-6 producing monocytes on stimulation with endogenous (LPS and TNC) and exogenous (TNC and MRP8) TLR ligands in SpA and ERA patients. 106 SFMC/ml in complete culture medium was used. [file 12969_2020_403_MOESM6_ESM.docx]

**Additional file 6:** **TNF^+^ and IL-6^+^ monocytes after stimulation with TLR ligands (LPS, PG, TNC and MRP8) in SFMC in SpA and ERA patients**

|  | SpA (n=10) | ERA (n=10) | p value |
| --- | --- | --- | --- |
| TNF^+^ monocytes (%) | | |  |
| *Unstimulated* | *35.25 (5.8)* | *45.3 (8.17)** | *0.0041* |
| *LPS stimulation* | *92 (32.55)* | *90.7 (18.71)* | *ns* |
| *PG stimulation* | *93.3 (21.3)* | *88 (22.3)* | *ns* |
| *TNC stimulation* | *70.35 (29.21)* | *65.8 (22.98)* | *ns* |
| *MRP8 stimulation* | *73.1 (3.53)* | *69.15 (35.73)* | *ns* |
| IL-6^+^ monocytes (%) | | |  |
| *Unstimulated* | *2.18 (1.51)* | *6.45 (3.95)** | *0.0002* |
| *LPS stimulation* | *28.3 (6.7)* | *26.45 (7.7)* | *ns* |
| *PG stimulation* | *28.3 (1.35)* | *27.4 (8.23)* | *ns* |
| *TNC stimulation* | *8.3 (2.26)* | *15.4 (10.28)** | *0.0074* |
| *MRP8 stimulation* | *10.4 (3)* | *20.95 (11.1)** | *0.0007* |

Results are expressed as median (IQR), * p <0.05. Exact p values are given in figure 5. *SpA:* Spondyloarthropathy; *ERA:* Enthesitis related arthritis
